# Development of Snake Fungal Disease after Experimental Challenge with *Ophidiomyces ophiodiicola* in Cottonmouths (*Agkistrodon piscivorous*)

**DOI:** 10.1371/journal.pone.0140193

**Published:** 2015-10-15

**Authors:** Matthew C. Allender, Sarah Baker, Daniel Wylie, Daniel Loper, Michael J. Dreslik, Christopher A. Phillips, Carol Maddox, Elizabeth A. Driskell

**Affiliations:** 1 Wildlife Epidemiology Lab, Department of Comparative Biosciences, College of Veterinary Medicine, University of Illinois, Urbana, IL, United States of America; 2 Illinois Natural History Survey, Prairie Research Institute, Champaign, IL, United States of America; 3 Department of Pathobiology, College of Veterinary Medicine, University of Illinois, Urbana, IL, United States of America; The University of Texas Arlington, UNITED STATES

## Abstract

Snake fungal disease (SFD) is a clinical syndrome associated with dermatitis, myositis, osteomyelitis, and pneumonia in several species of free-ranging snakes in the US. The causative agent has been suggested as *Ophidiomyces ophiodiicola*, but other agents may contribute to the syndrome and the pathogenesis is not understood. To understand the role of *O*. *ophiodiicola* in SFD, a cottonmouth snake model of SFD was designed. Five cottonmouths (*Agkistrodon piscivorous*) were experimentally challenged by nasolabial pit inoculation with a pure culture of *O*. *ophiodiicola*. Development of skin lesions or facial swelling at the site of inoculation was observed in all snakes. Twice weekly swabs of the inoculation site revealed variable presence of *O*. *ophiodiicola* DNA by qPCR in all five inoculated snakes for 3 to 58 days post-inoculation; nasolabial flushes were not a useful sampling method for detection. Inoculated snakes had a 40% mortality rate. All inoculated snakes had microscopic lesions unilaterally on the side of the swabbed nasolabial pit, including erosions to ulcerations and heterophilic dermatitis. All signs were consistent with SFD; however, the severity of lesions varied in individual snakes, and fungal hyphae were only observed in 3 of 5 inoculated snakes. These three snakes correlated with post-mortem tissue qPCR evidence of *O*. *ophiodiicola*. The findings of this study conclude that *O*. *ophiodiicola* inoculation in a cottonmouth snake model leads to disease similar to SFD, although lesion severity and the fungal load are quite variable within the model. Future studies may utilize this model to further understand the pathogenesis of this disease and develop management strategies that mitigate disease effects, but investigation of other models with less variability may be warranted.

## Introduction

Emerging wildlife diseases are increasing in prevalence worldwide, and can have dramatic impacts on local populations [1]. Snake fungal disease (SFD) is one such disease syndrome associated with high morbidity and mortality in free-ranging snakes, predominantly eastern massasaugas (*Sistrurus catenatus*) and timber rattlesnakes (*Crotalus horridus*) [2–9]. Clinical signs in eastern massasaugas have commonly involved facial disfiguration associated with the nasolabial pits leading to a high mortality[2]. The causative agent of this disease has been linked to *Ophidiomyces ophiodiicola* [10], but has not been confirmed with experimental challenge studies. Furthermore, other fungal organisms have been present in these cases, thereby raising doubt that *O*. *ophiodiicola* is the sole or primary pathogen involved in SFD [10–12].

A model is needed to characterize the pathogenesis of SFD through experimental challenge. Experimental transmission studies are historically used in model species to demonstrate the ability of a pathogen to study a disease syndrome in threatened species [13]. Determining causation through Koch’s postulates is paramount to characterizing and studying the disease syndrome. The eastern massasauga and timber rattlesnake are both are under local threats to their sustainability, thus studying SFD in these target species is limited to observational studies. However, cottonmouths (*Agkistrodon piscivorous*) are an abundant pit-viper species overlapping in distribution with both the eastern massasauga and timber rattlesnake.

Therefore, an experimental model for SFD in cottonmouths was designed to test the following hypotheses: 1) Experimental inoculation of *O*. *ophiodiicola* would result in a disease syndrome consistent with SFD that is observed through clinical signs and histopathology; and 2) Two assay methods, nasolabial swabs, and saline flushes, would provide equal qPCR detection of *O*. *ophiodiicola*.

## Materials and Methods

### Animals and husbandry

Sample size was determined using the following *a priori* information: alpha = 0.05, power = 0.8, and 75% of the inoculated animals would be infected and a single control animal would be uninfected within the same trial. Six adult cottonmouths (*Agkistrodon piscivorous*; 3 males and 3 females) were collected via visual encounter surveys from a large, wild population in southern Illinois and acclimated to room temperature between 25–27°C for two weeks. Five animals (3 males and 2 females) were randomly selected as experimentally challenged animals using a random number generator (treatment group) whereas one animal (female) was maintained as a control (mock-infected). Treatment and control animals were housed singly in 20 gallon enclosures (Neodesha Plastics Inc., Neodesha, Kansas) in separate 15’9” x 12’ environmental chambers (Rheem Puffer Hubbard, Atlanta, Georgia) maintained at 25–27°C. All individuals were bedded on newspaper and provided water *ad libitum*. Pre-killed mice were offered approximately every 1–2 weeks.

A physical examination was performed on each individual upon presentation and prior to inoculation and deemed to be free of clinical signs. After inoculation, clinical signs were scored as absent (0) or present (1) daily, including lethargy, facial swelling, open-mouth breathing, skin lesions (other than the head), and discharge from the face.

Endpoints for euthanasia established for snakes included: Weight loss of greater than 10% in a one week period, extreme lethargy, lack of righting reflex, granuloma growth that prevents eating, drinking, or visual activity. The animals were evaluated for clinical signs once to twice daily, there were no unexpected deaths. Snakes were euthanized by complete anesthesia with ketamine intramuscularly, followed by sodium pentobarbital intravenously. Analgesics were not administered during the study due to the unknown nature of how that will affect disease progression. We need to establish realistic criteria for free-ranging animals to maximize the usefulness of this study. The endpoint criteria were established to minimize pain and discomfort and euthanasia was then performed.

### Fungus preparation

The fungal isolate (UI-VDL # 12–34933) was cultured from an eastern massasauga in 2012 and stored on a potato dextrose agar slant at -80°C. The isolate was propagated on sabaroud dextrose agar at 25°C for 10 days. Conidia were dissociated from the hyphae by vortexing a saline suspension of mycelium then filtering the material through sintered glass wool to remove the hyphae. The number of colony forming units in the suspension was approximately 10^7^ viable conidia per ml as estimated by hemacytometer count and confirmed by plating serial dilutions.

### Animal Inoculation and assay methods

All snakes tested negative for *O*. *ophiodiicola* using qPCR (on days -7 and -14 of the study) and were free of clinical disease. Both nasolabial pits of treatment snakes were inoculated with 0.1 ml of a pure culture containing 109,000 colony forming units of *O*. *ophiodiicola* using a polypropylene catheter (Argyle open end catheter, Covidien, Mansfield, MA, USA). The control (mock-infected) animal was inoculated with a similar volume of sterile saline into each pit. The nasolabial pits were selected because clinical disease in massasaugas are frequently associated with this site.

**Fig 1:**
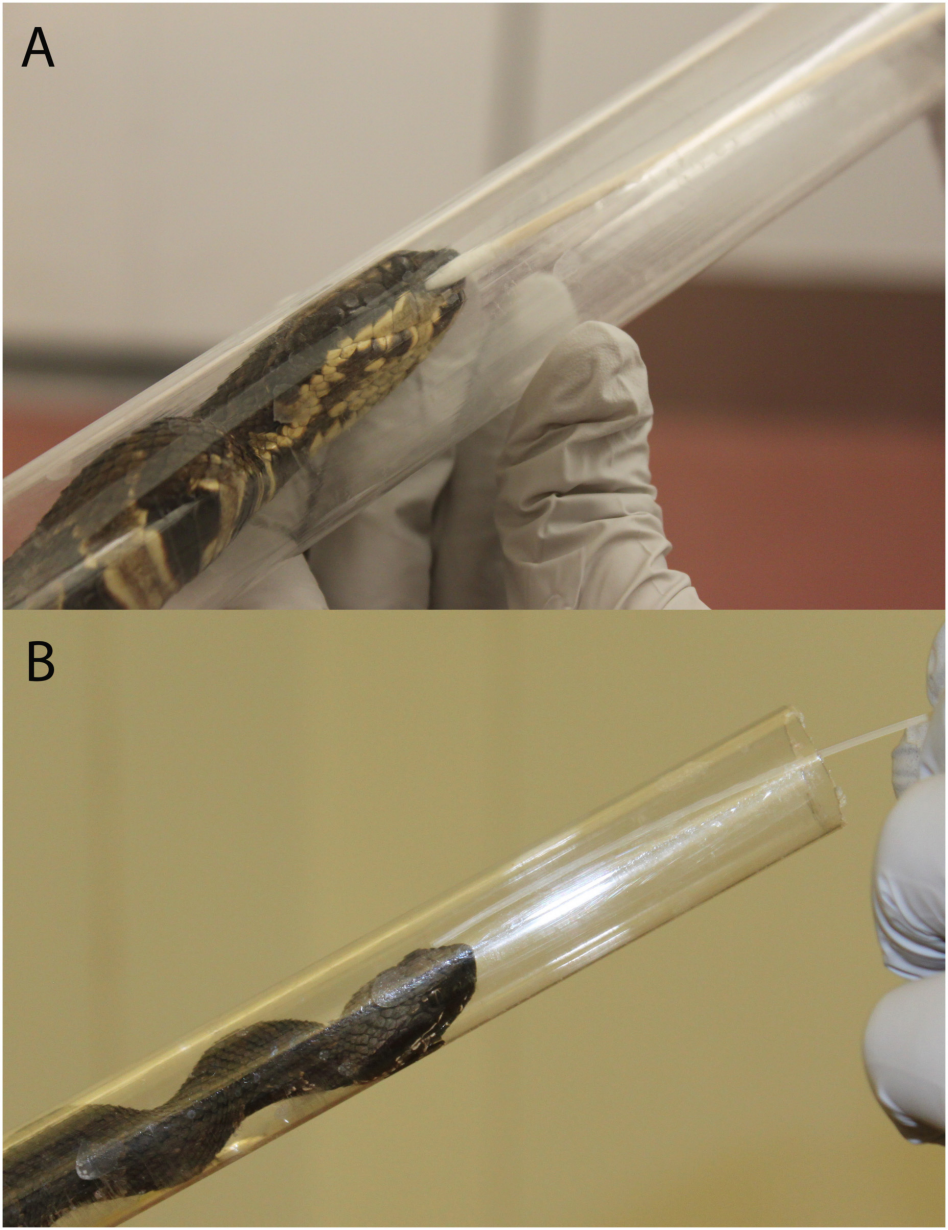
Sample collection for Snake Fungal Disease. Collection of nasolabial swab (A) and flush (B) samples from cottonmouth (*Agkistrodon piscivorous*) experimentally challenged with *Ophidiomyces ophiodiicola*.

**Fig 2:**
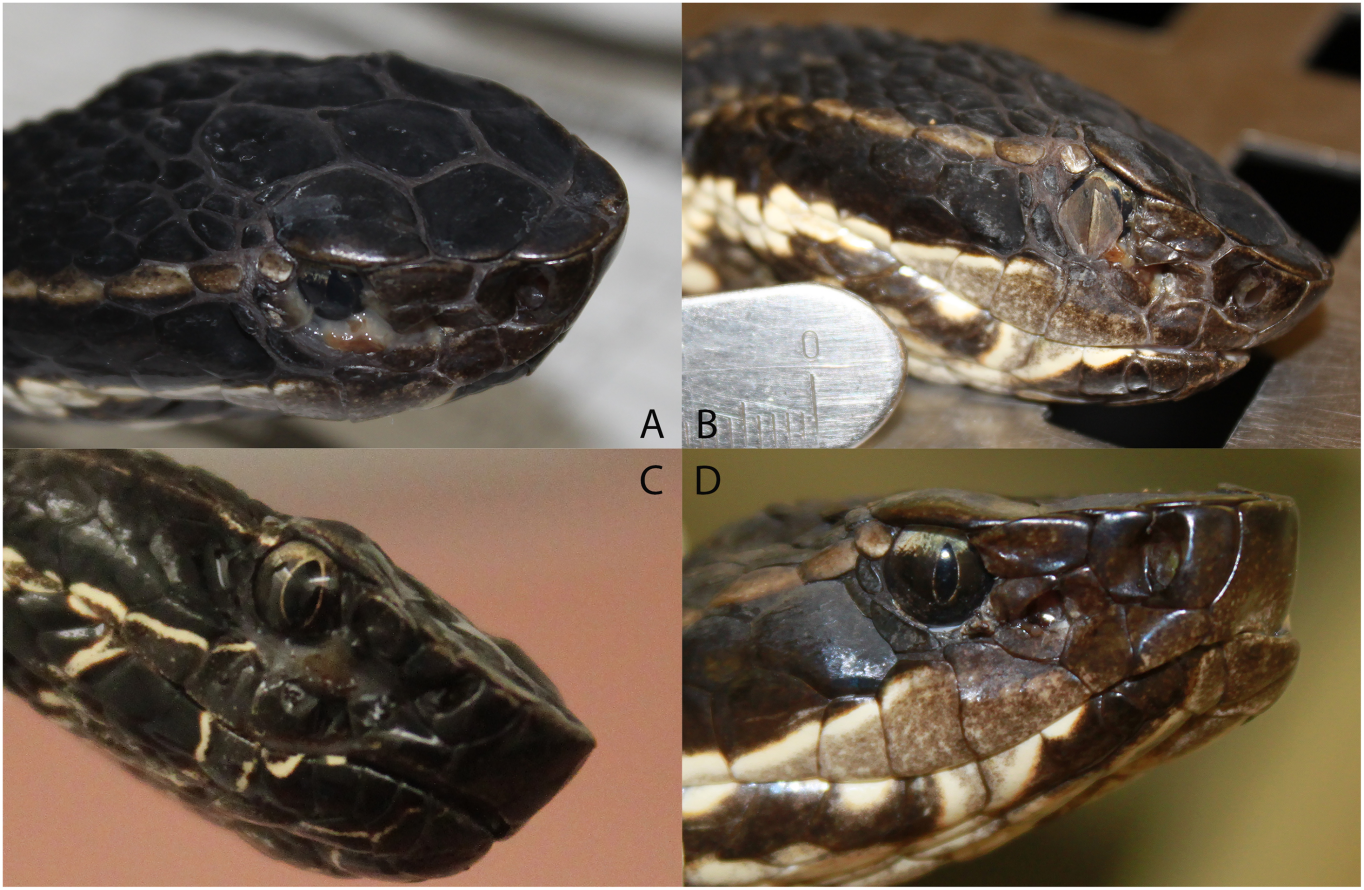
Facial swelling in Cottonmouths with Snake Fungal Disease. *Facial swelling observed in cottonmouths (Agkistrodon piscivorous) experimentally challenged with Ophidiomyces ophiodiicola. Severity ranged from severe (A, B), moderate (C), and mild (D)*.

Samples from each nasolabial pit were collected twice weekly (on the same day) for 90 days after challenge. Two sampling methods were used for each snake: 1) direct collection of epithelial cells in the right nasolabial pit using cotton-tipped swabs (Fig 1A) and 2) saline flushes of the left nasolabial pit using the polypropylene catheter and subsequent collection of the aspirated material (Fig 1B).

DNA was extracted from all swabs and flushes using the DNAeasy blood and tissue kit (Qiagen Inc., Valencia, CA) with the additional step utilizing lyticase [14]. All DNA samples were measured to determine DNA concentration and purity (A260/280 ratio) using spectrophotometry. All samples were confirmed to have reptile DNA targeting mitochondrial markers in conventional PCR assays [14]. The sampling technique that resulted in the highest DNA quantity and purity was chosen as the final sample type for qPCR analysis. Fresh tissue of the nasolabial pit collected at necropsy were ground using a bead beater in a volume of 180 μl RLT buffer to which 12μl of 25 U/μl of lyticase was added then incubated at 37°C for 1 hour. DNA was extracted following manufacturer’s instructions (DNAeasy blood and tissue kit, Qiagen). Finally, each sample was assayed using qPCR targeting a 68 basepair segment of the ITS gene segment [14].

### Pathology

A full necropsy was performed on all study animals. Aseptic procedures were used to collect fresh tissue samples from the nasolabial pits and skin lesions for fungal culture and qPCR. Another set of tissues (head, skin lesions, lung, liver, splenopancreas, kidney, and intestine) were collected and placed in 10% neutral buffered formalin for histopathologic examination. Fixed samples were routinely processed for light microscopy. Briefly, sections of formalin-fixed, paraffin-embedded tissues were deparaffinized, sectioned at 3 μm, and stained with hematoxylin and eosin. Additional sections of the head at the nasolabial pits and of skin lesions were also stained with Gomori methenamine silver (GMS) stain to identify the presence of fungal hyphae. The histopathology was performed by a single co-author (EAD).

### Statistical analysis

Binomial prevalence estimates (based on qPCR and histopathology) and overall survival was computed. Life tables were constructed for days to death (median survival time) in inoculated snakes, and those individuals that survived to the completion of the study were censored on the day that study ended (day 90). To determine if the presence/absence of clinical signs were associated with time since inoculation, a mixed-effects binary logistic regression with individual assessments as the random effect was used. Descriptive statistics (median, 10–90 percentile) were calculated for DNA concentration and purity (A260/A280 ratio) from swab and flush samples for each timepoint and compared using a Friedman’s test for repeated measures of non-normally distributed data. Concentration or purity within each sample type that were not significantly different over time were combined and evaluated using a Mann-Whitney U test. Sensitivity and specificity of the qPCR assay in both swab and necropsy samples was determined compared to histopathological evidence of inflammation and presence of fungal hyphae. Statistical significance was considered for all p values ≤0.05. All analysis was performed using commercial software (SPSS 22, IBM statistics, Chicago, IL).

## Results and Discussion

### Survival

Two treatment snakes died during the 90 days of the study (day 35 and day 86), while three survived until euthanasia on day 90, resulting in a 40% mortality rate. Each snake that died prior to the conclusion of the study had clinical and histopathological signs consistent with SFD, and/or positive qPCR results. The control snake (AP-01) developed a severe infection in its tail around day 20, which was attributed to the frequent blood draws (associated with a concurrent study), and was euthanized on day 31. Thus, survival comparisons could not be made between treatment and control group. Because most animals survived to completion of the study, the median survival time was calculated at 90 days, but likely would have been longer if surviving animals were not censored.

### Clinical signs

**Fig 3:**
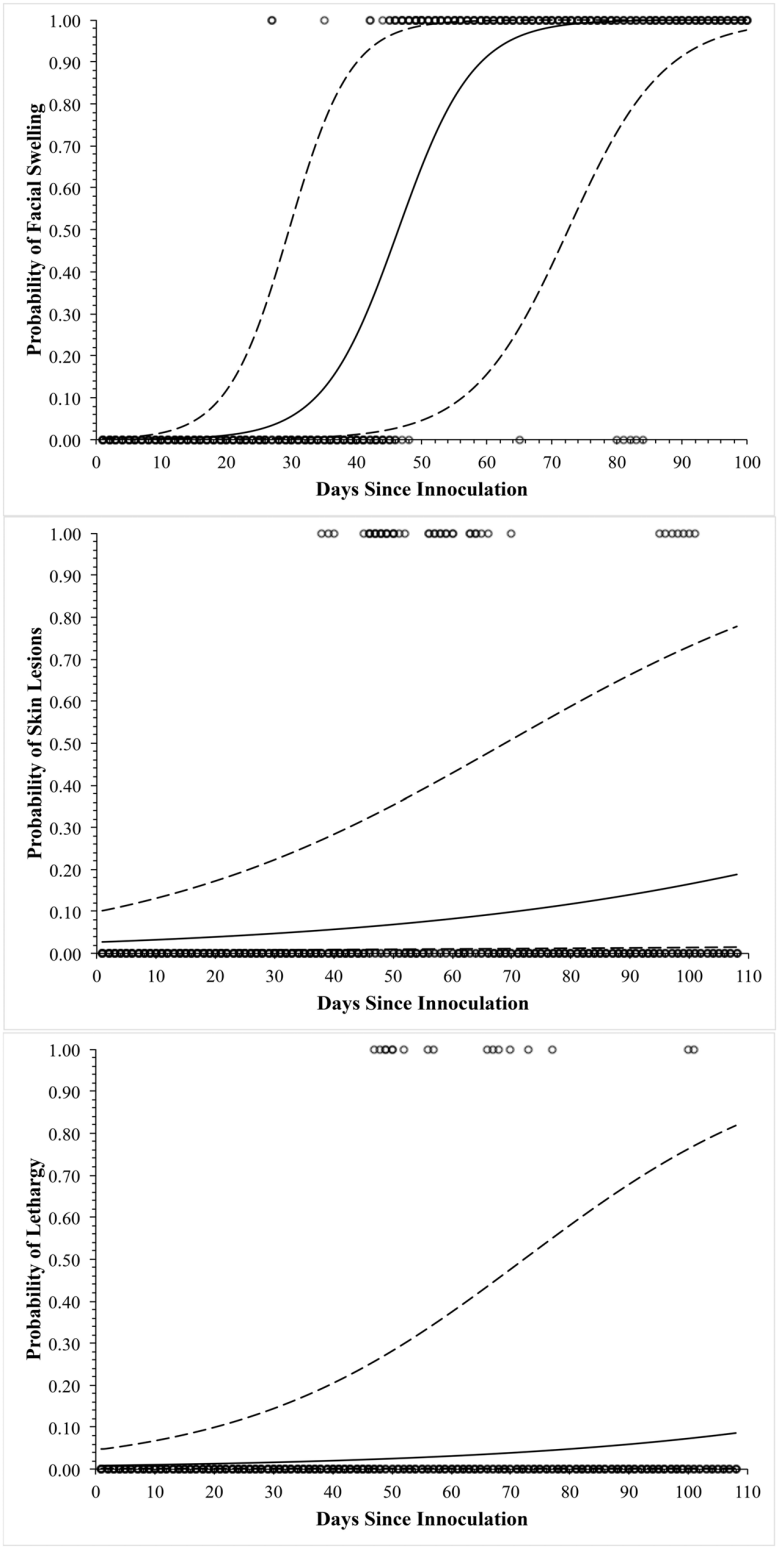
Clinical signs of Snake Fungal Disease. Graphs demonstrating the probability of facial swelling (A), skin lesions (B), and lethargy (C) in cottonmouths (*Agkistrodon piscivorous*) after experimental inoculation of the nasolabial pit with *Ophidiomyces ophiodiicola*. Solid line indicates the estimate, dashed lines indicate the 95% confidence interval, and open circles represent the presence or absence of the clinical sign.

**Fig 4:**
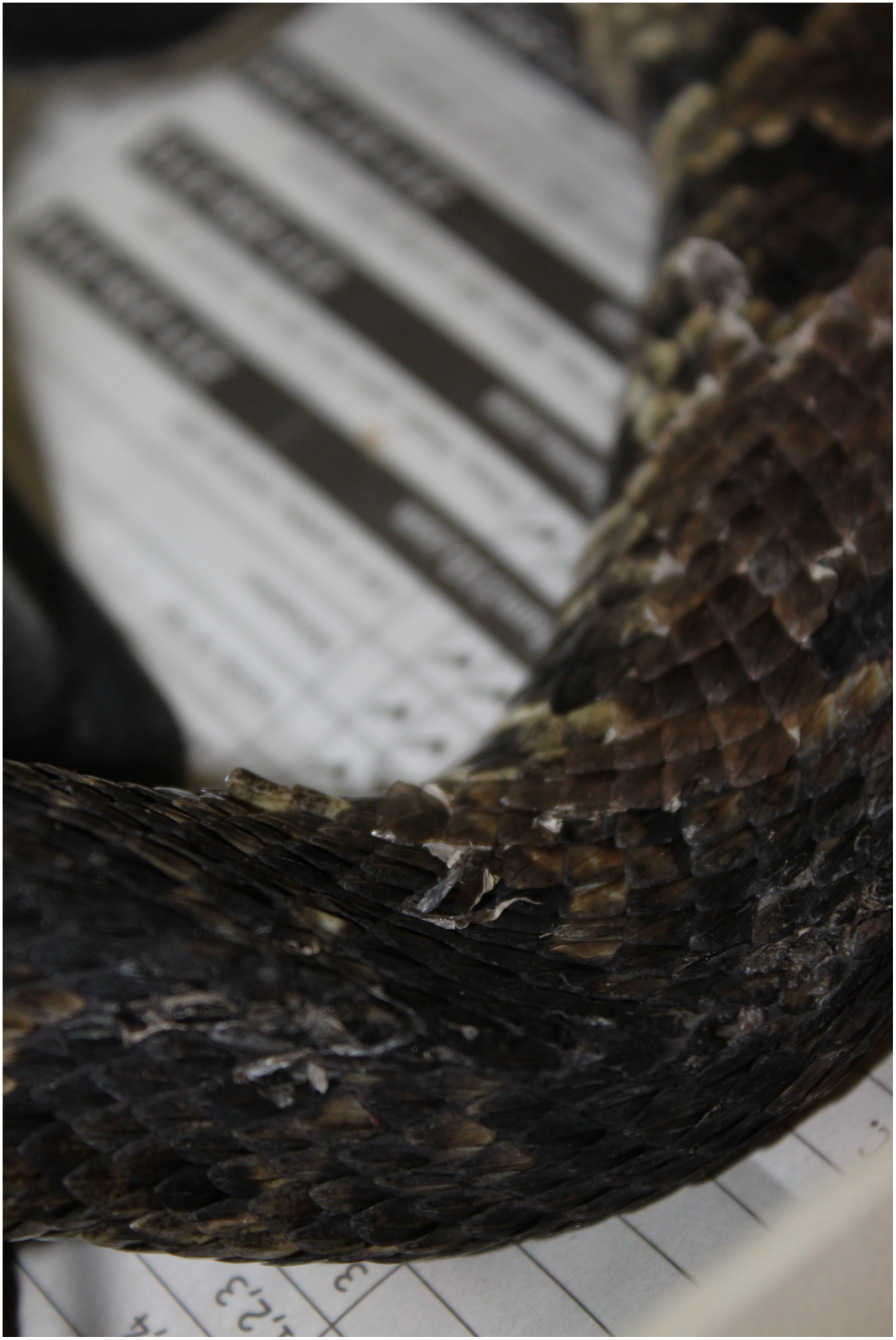
Skin lesions in cottonmouths with Snake Fungal Disease. *Non-facial skin lesions observed in a cottonmouth (Agkistrodon piscivorous) experimentally challenged with Ophidiomyces ophiodiicola*.

Only facial swelling (p<0.001) significantly differed over time and was present in the greatest proportion of snakes (Figs 2 and 3A). Non-facial skin lesions were observed in three individuals (Fig 4). Open-mouth breathing was observed too infrequently for analysis. Clinical signs associated with infection were initially observed on day 12, with most snakes showing signs by days 30–37 (Fig 3). Non-facial skin lesions (Fig 3B) and lethargy (Fig 3C) were seen infrequently and did not differ over time.

### Pathology

The negative control subject (AP-01) had a mild serocellular crust present in one nasolabial pit, possibly caused by sampling procedures. The tail had a region of necroulcerative dermatitis associated with numerous bacteria and vertebral osteonecrosis; this was attributed to the infection which developed secondary to repeated venipuncture. Microscopic sections of both the head and the tail lesion were examined with a GMS stain and no fungal hyphae were observed.

**Fig 5:**
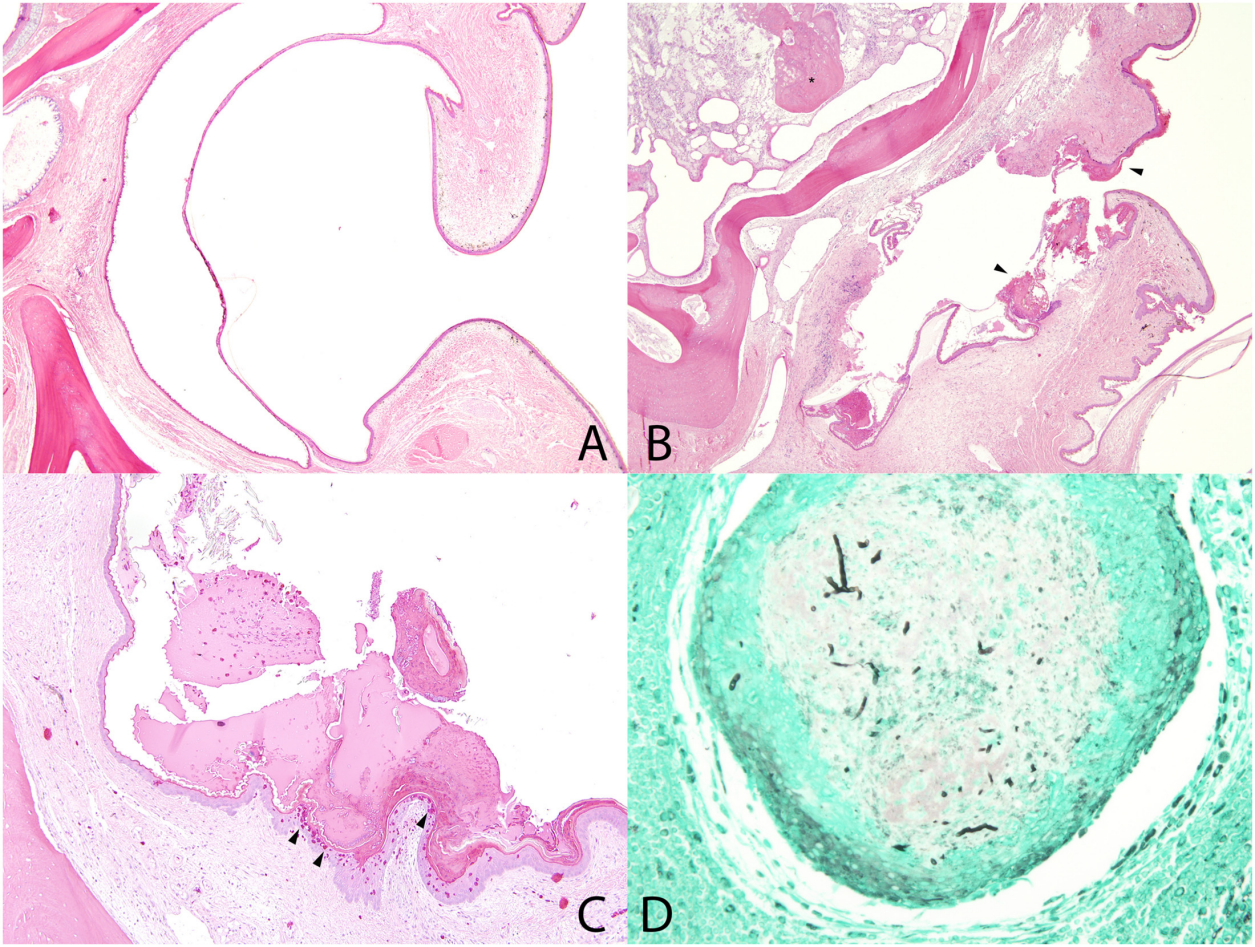
Snake Fungal Disease Histopathology. Microscopic lesions in cottonmouth snakes inoculated with *Ophidiomyces ophiodiicola*. A. Normal nasolabial pit. HE stain. B. Nasolabial pit with epithelial thickening and crusts (arrowheads), erosion, ulceration, and dermatitis. Deeper in the head, there is osteomyelitis (asterisk). HE stain. C. Nasolabial pit with crusts and heterophilic dermatitis (arrowheads). HE stain. D. Granuloma deep to the nasolabial pit that contains intralesional fungal hyphae (black linear branching). GMS stain.

The five study animals inoculated with *O*. *ophiodiicola* exhibited similar unilateral lesions in the region of the nasolabial pit varying in severity. All inoculated snakes had heterophilic dermatitis with varying degrees of erosion to ulceration in the region of the pit, with a mild to abundant serocellular crust covering the area (Fig 5B and 5C). Some snakes (AP-02 and AP-03) also had an extension of the nasolabial pit lesion into the deeper tissues with a heterophilic fasciitis, myositis, and osteomyelitis. There were heterophilic granulomas present in the deeper tissues and bone of two snakes (AP-02 and AP-05;Fig 5B). All snakes had abundant bacterial colonies associated with these lesions. Snakes AP-04 and AP-06 had mild, small lesions where snakes AP-02, AP-03, and AP-05 all had more severe lesions that extended to the deep tissues and bone, with snake AP-02 demonstrating the most extensive lesions. GMS stained sections of the head revealed presence of small numbers of 3–5 μm in diameter fungal hyphae with parallel walls in snakes AP03, AP-05, and AP-06 (Fig 5D). Multiple sections of the head (up to seven sections) were examined in all snakes, but no fungal hyphae were observed in snakes AP-02 or AP-04 despite the presence of similar nasolabial pit lesions. Snake AP-02 also had a similar ulcerative heterophilic dermatitis lesion on the body wall skin; however, no fungal hyphae were observed in this lesion with GMS stain.

There were minimal lesions in other examined tissues. Snake AP-06 had renal and hepatic thrombi with bacterial bacilli, suggestive of sepsis. Most snakes had a variety of different parasites present. All snakes except AP-03 had hemogregarine coccidial schizonts (likely *Hepatozoon sp*.) within the lung. Snakes AP-01, AP-02, AP-03, and AP-04 had larval trematodes (mesocercaria) within the visceral adipose and/or liver, and snakes AP-02, AP-03, and AP-05 also had adult trematodes (likely renifers) within the faveolar spaces of the lung and/or in the oral and nasal cavity. Finally, there was also the presence of cestodes and/or migrating mural nematodes within the intestine of snakes AP-01, AP-03, and AP-06. Snake AP-03 also had an amyloid-like material filling hepatic sinusoids.

### Assay Methods

DNA concentrations from swabs (median: 3.5 ng/μl; 10–90 percentiles: 0–13.9) were significantly higher than saline flushes (median: 0 ng/μl; 10–90 percentiles: 0–0.6; p<0.0001). Mitochondrial DNA generic to reptile DNA was consistently present in swab samples, but not flush samples, indicating that flushes were an insufficient means to acquire usable DNA. Thus quantitative polymerase chain reaction (qPCR) was performed only on swab samples. Quantitative PCR detected *O*. *ophiodiicola* DNA in each of the inoculated snakes. *Ophidiomyces ophiodiicola* DNA was never detected in the control snake (Table 1). All treatment snakes were positive on day 3 post-inoculation, and all swabs were negative after day 58 (Table 1). The peak median fungal DNA quantity was observed on day 7, with a secondary peak on day 19. The highest single quantity of *O*. *ophiodiicola* DNA was observed on day 19 with 26,354 copies. The duration each snake was qPCR positive was variable, ranging from 3 to 55 days (Table 1). Swab samples were 100% sensitive for detection of *O*. *ophiodiicola* DNA at some point during for which the study, but 0% sensitive when comparing the last swab sample collected prior to death with histopathologic evidence of fungal hyphae. Fresh tissue from the nasolabial pits tested positive for the presence of *O*. *ophiodiicola* DNA in three treatment animals, for which fungal hyphae were also detected by histopathology. The qPCR assay was 100% sensitive and 100% specific at necropsy when comparing to histopathologic evidence of disease.

**Table 1:** Summary of survival, DNA detection from swabs, and presence of microscopic lesions consistent with SFD in cottonmouth snakes (*Agkistrodon piscivorous*) experimentally challenged with *Ophidiomyces ophiodiicola*. AP-01 (mock-inoculated control snake) died from an infection of the tail secondary to venipuncture. ND = not detected.

Individual	Sex	Survival	Day of Death	Peak DNA quantity qPCR swab	First positive qPCR swab	Last positive qPCR swab	Microscopic lesions of SFD	Fungal hyphae observed microscopically	Nasolabial tissue qPCR
AP-01 (mock-inoculated)	Female	No	31	NA	NA	NA	No	No	No
AP-02	Male	No	86	Day 7	Day 3	Day 7	Yes	No	No
AP-03	Male	Yes	NA	Day 19	Day 3	Day 58	Yes	Yes	Yes
AP-04	Female	Yes	NA	Day 3	Day 3	Day 22	Yes	No	No
AP-05	Male	Yes	NA	Day 7	Day 3	Day 37	Yes	Yes	Yes
AP-06	Female	No	35	Day 3	Day 3	Day 3	Yes	Yes	Yes

This study demonstrated cottonmouths experimentally challenged with *O*. *ophiodiicola* produced clinical, molecular, and histopathologic evidence of SFD. Previous reports have shown at least two species of pit-vipers to be observed with SFD and *O*. *ophiodiicola* has been associated with lesions [2–5,8–11,15], but causation was never described until the present study. While disease was induced in cottonmouths with *O*. *ophiodiicola*, improvements to the model are needed to produce clinical disease similar to what has been observed in affected free-ranging eastern massasaugas and timber rattlesnakes.

Survival from SFD is low, yet variable depending on species and presentation [2,4,5, 10,16–17]. The current study found a 40% mortality rate, lower than is observed in eastern massasaugas [2,10], the species from which this isolate originated. The lower mortality rate in this experimental model may be due to the difference in pathogenicity based on species, temperature, route of exposure, time, or environmental variables. timber rattlesnakes in the Northeast US had a 17% mortality rate, and they attributed that to an anesthetic complication [5]. However, that study did not follow the snakes indefinitely and all were lost to follow-up, which may have underestimated mortality rate [5]. Additionally, it is possible that eastern massasaugas developing less severe disease and/or clearing the disease were not sampled during mark-recapture studies [2,3]; therefore the true mortality rate in the wild was overestimated. The median survival time of 90 days is a reflection of the majority of cottonmouths surviving to the end of the study. The time to death in natural cases of disease is unknown, but has been reported to be as short as 21 days [2] or as long as 155 days [5], but these were measured after clinical signs were already reported. The course of disease in natural cases is likely influenced by physiological, environmental, and genetic factors. Growth of the fungus is enhanced at certain temperatures, substrates, and pH *in vitro* [10], therefore further challenge studies utilizing this model may help to elucidate the environmental factors in combination with the animal responses.

Facial swelling is a common sign in many case reports of SFD [2,4,5,11] and was the most common clinical sign observed in this study. The nasolabial pits have been proposed as an entrance site for *O*. *ophiodiicola* [3] and thus utilized in this model. Facial swelling was expected at the inoculation site; however, the severity was less than in natural infections. The severity of the lesion may vary with challenge strain or host immune response. Facial swelling in treatment animals was exclusively observed on the right side of the face, where swabbing occurred. It is possible infection only occurred on the right side because the swab technique caused a break in the natural epithelial barrier, thus allowing *O*. *ophiodiicola* to invade underlying tissue. The left nasolabial pit was sampled exclusively using a saline flush, which may have produced less physical irritation and inflammation than swabbing, thus resulting in failure of *O*. *ophiodiicola* to establish an infection on this side. This finding suggests pathogenesis requires the disruption of the epithelial barrier to establish an infection. Thus, the model better represents natural infections if physical abrasion occurred prior to the introduction of the fungal organisms.

The lesions present in the inoculated snakes are similar to lesions observed in natural cases [2]. However, natural cases in eastern massasaugas typically have abundant fungal hyphae present within the lesion. Fungal hyphae were difficult to detect in inoculated snakes, with small numbers of intralesional fungal hyphae only observed in three of five animals microscopically, despite extensive sectioning. Bacteria are often found in conjunction with the lesions in eastern massasaugas and speculated to be secondary to the extensive cutaneous ulceration, which were also observed in this study. Two of the cottonmouths with longest history of positive qPCR results for *O*. *ophiodiicola* also had the most severe microscopic lesions (AP-03 and AP-05).

After inoculation, treatment snakes demonstrated the presence of *O*. *ophiodiicola* DNA for up to 58 days. However, the presence of clinical signs did not mirror DNA results, as clinical signs first occurred on day 13, and were not consistently observed until day 31, by which time all but two individuals had negative qPCR results. This pathogen has been shown to be keratinophilic and invasive to tissues [2,10]. It is possible that while this pathogen is on the surface of the nasolabial pit, it can be detected, but has not yet become invasive. Thus, clinical signs may not occur until the epithelium is invaded and tissue architecture disrupted, by which point sampling with cotton-tipped applicators may yield false negative results. Biopsies or firmly swabbing may be more sensitive in detecting *O*. *ophiodiicola* than routine sampling, and thus have been recommended in other studies despite a 2% false negative rate with those tissues as well [15]. However, surgical biopsies in small snakes or individuals in the field are impractical. Furthermore, this may demonstrate a failure of the model to represent natural infection, and paired biopsies and swabs in snakes with natural infection are needed to determine the accuracy of the sampling method.

The use of a swab produced superior DNA quantity and quality compared to a saline flush of the nasolabial pit. *Ophidiomyces* DNA from swabs have been amplified in surveys of free-ranging snakes [14], but their utility had not been fully evaluated. Swabs were able to detect shedding of this pathogen in all animals for up to 58 days after inoculation, thus resulting in 100% sensitivity at some point during the course of disease. However, there was histological evidence of fungal pathogens in only three of the five animals, which comprised the animal that died first (AP-06) and the two animals that were shedding *O*. *ophiodiicola* the longest (AP-03 and AP-05). When comparing histopathologic presence of fungal hyphae to qPCR at necropsy, the assay was also 100% sensitive and 100% specific, but 0% sensitive in detecting the DNA in the swab sample immediately prior to death. The snakes that stopped shedding by days 7 (AP-02) and 22 (AP-04) had no evidence of the fungus at the time of death by molecular testing and microscopic examination. It is possible these individuals cleared the infection. Alternatively, it was not uncommon to find rare fungal hyphae microscopically by extensively examining numerous tissue sections. While this fungus has shown to be locally invasive in multiple studies [2,15], the abundance of *O*. *ophiodiicola* in tissue in this model was highly variable, highlighting the importance of evaluating multiple sections of tissues using histopathology and performing molecular diagnostics to ensure detection.

## Conclusions

Cottonmouth snakes inoculated with *O*. *ophiodiicola* showed clinical signs and histopathological evidence consistent with SFD. These findings validate cottonmouths are susceptible to SFD and further supports *O*. *ophiodiicola* plays a role in SFD. Unilateral lesion development in this study suggests the disruption of the epidermal barrier may be a key component to the establishment of infection with *O*. *ophiodiicola*. In this model, the number of *O*. *ophiodiicola* organisms in the skin lesions was low based on molecular and microscopic findings. Swabbing facial lesions was a viable sampling method to detect DNA early in infection and for up to two months after inoculation. However, during later stages, when the pathogen invades tissue, swabs are not likely an effective means of detection. While cottonmouths were chosen for this study as a comparable species to the imperiled eastern massasauga and timber rattlesnakes, further work is needed to develop this species as a suitable model for this disease. This study has provided a framework for a model for SFD, which will aid in determining the pathogenesis of this disease.

### Ethics Statement

All activities for this study were specifically approved by the University of Illinois Institutional Animal Care and Use Committee (Protocol: 12199). The University of Illinois is registered with the USDA and has an assurance on file with OLAW. A collection permit was issued by the Illinois Department of Natural Resources under permit 05-11S.

## Supporting Information

S1 File Raw Data. (XLSX)
